# The myth of antibiotic spider silk

**DOI:** 10.1016/j.isci.2021.103125

**Published:** 2021-10-05

**Authors:** Simon Fruergaard, Marie Braad Lund, Andreas Schramm, Thomas Vosegaard, Trine Bilde

**Affiliations:** 1 Interdisciplinary Nanoscience Center, Department of Chemistry, Aarhus University, Aarhus, Denmark; 2 Department of Biology, Aarhus University, Aarhus, Denmark

**Keywords:** Entomology, Bacteriology

## Abstract

Spider silk is frequently attributed antimicrobial properties. This notion is based on studies reporting antimicrobial activity (AMA) of spider silk; however, close inspection of these studies reveals that the evidence is conflicting, and at best anecdotal. We performed a systematic study of antimicrobial properties of different silk types from seven species across the spider phylogeny. We found no evidence of AMA of silk in direct contact and disc diffusion assays against Gram-negative *Escherichia coli* and *Pseudomonas putida*, and the Gram-positive *Bacillus subtilis*. Furthermore, staining experiments and fluorescence microscopy showed the presence of live bacteria on silk surfaces indicating no antimicrobial effect on direct contact. A critical evaluation of the literature reveals that published tests of AMA are scarce and that all the studies claiming positive results are compromised by methodological shortcomings. Our analysis demonstrates that the common notion that spider silk is antimicrobial is not supported by empirical data.

## Highlights

• Tests of silk from 7 spider species revealed no evidence for antimicrobial activity AMA
• Reports of AMA of spider silk are compromised by inadequate controls or contamination
• Published reports of AMA cannot be unequivocally attributed to spider silk
• The notion of inherent antimicrobial spider silk is not supported by empirical data

## Graphical abstract

**Figure:**
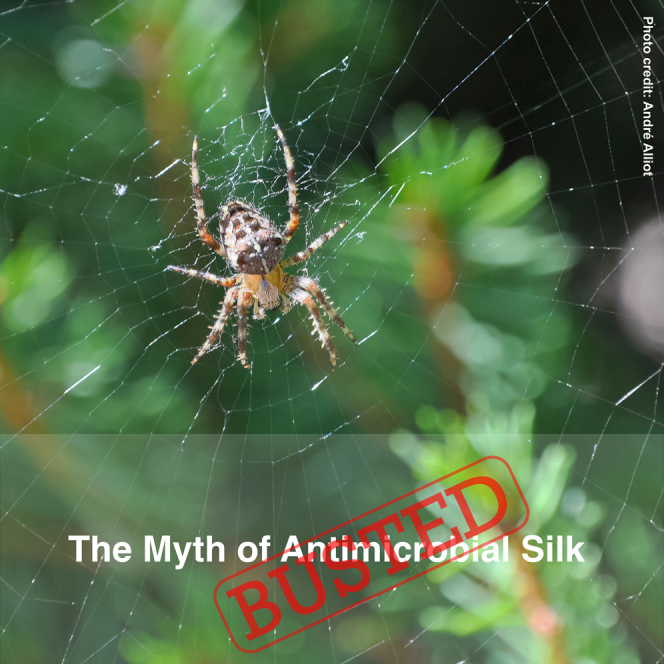

## Introduction

**Figure 1:**
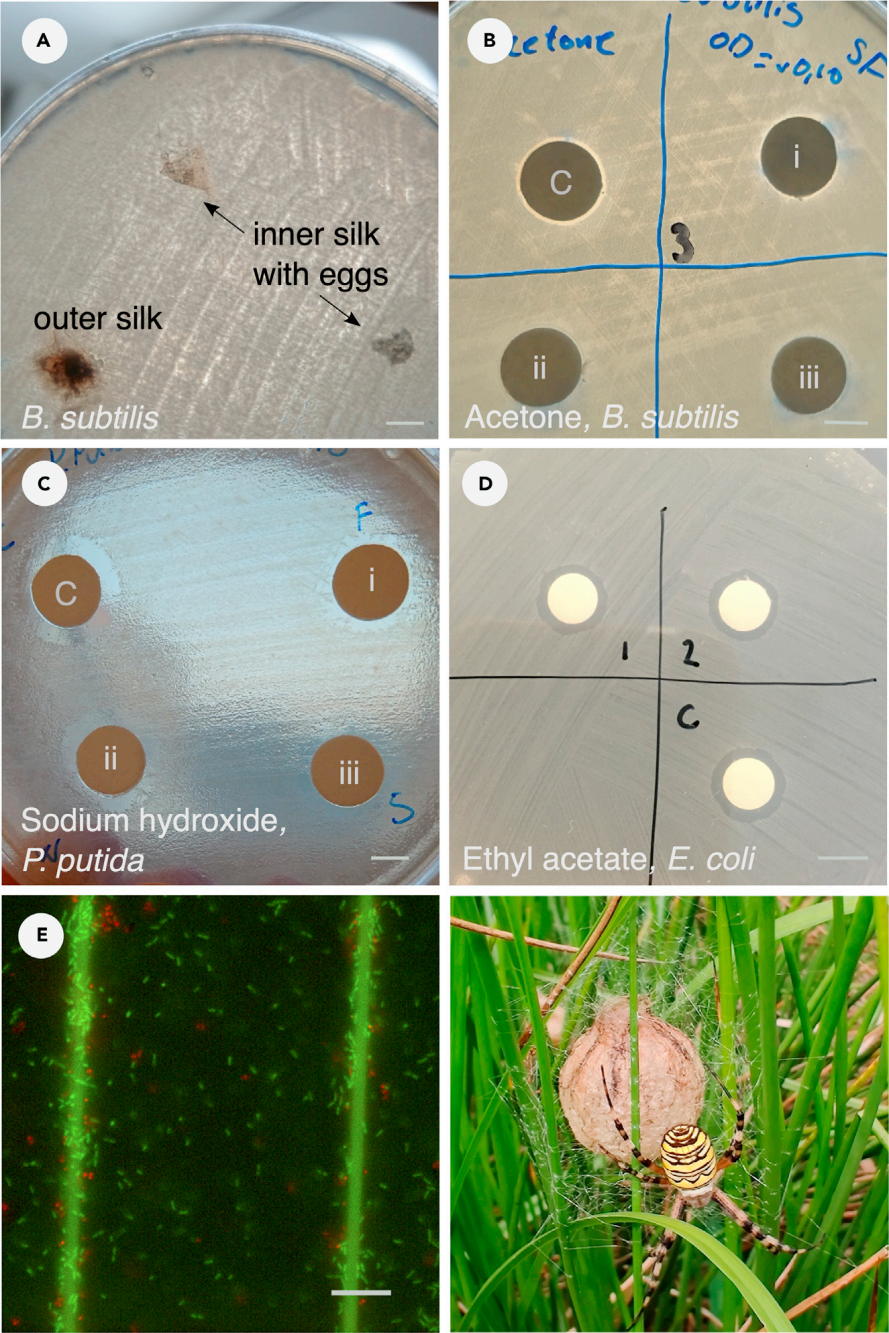
Tests of antimicrobial activity of spider silk (A) direct contact test of *A. bruennichi* outer and inner egg case silk against *B. subtilis*. (B–D) disc diffusion assay of silk extracted in acetone (B), sodium hydroxide (C), or ethyl acetate (D). Solvent control (C), *C. versicolor* burrow web (i), *N. edulis* orb web (ii), *N. edulis* dragline silk (iii), and *S. dumicola* capture web and nest silk (1, 2). (E) *E. coli* incubated for 3 h with *A. bruennichi* dragline silk; live (green) bacteria, and a few dead (red) bacteria, are located on silk fibers. Scale bar = 20 μm. The photo shows the spider *Argiope bruennichi* with egg case and web (Photo T. Bilde).

Spiders express extraordinary behavioral and physiological adaptations that enable them to occupy a very broad range of habitats. One of their most prominent adaptations is the production of silk, which is an extended phenotype involved in most aspects of spider biology. The silk consists of protein-based biopolymer fibers, and spiders produce different silk types that are task-specific (Vollrath, 2000). Spider silk is renowned for its extraordinary physical properties such as high tensile strength and flexibility (Vollrath, 2000), facilitating a variety of functions (Foelix, 2011). For example, silk is used as an anchor for rapid escape, a snare for prey capture, to immobilize cannibalistic mates, to make egg cases for protection (Figure 1 low left), in meter long lengths for parasailing, and even in silk diving bells that facilitate underwater life.

It has repeatedly been asserted that silk also provides an active defense against pathogens. Historically, spider silk was attributed healing properties and records report a variety of medical applications, in particular in relation to wound healing (Heimer, 1988; Newman and Newman, 1995). In this context, silk fibers are believed to possess antimicrobial properties, and studies reporting potential antimicrobial molecules on spider silk, such as metal chelators (Hu et al., 2007) and fatty acids (Hattori et al., 1987), have substantiated this notion (Romer and Scheibel, 2008; Saravanan, 2006). There are two main reasons why spider silk would benefit from antimicrobial properties: protecting the spider or protecting the silk itself. First, spiders are often found in silk lined retreats, e.g. underground, and their eggs are always deposited inside a silk case; therefore the deposition of antimicrobial molecules on the silk could function to protect against pathogens. Spider eggs consist of energy-rich compounds and water, which makes them an ideal substrate for pathogens and other microorganisms, therefore it is reasonable to hypothesize that egg case silk (cylindrical and aciniform silk) or the eggs themselves could possess antimicrobial properties (Babczynska et al., 2019; Makover et al., 2019). Second, spider silks are protein-based fibers with primarily nonpolar and hydrophobic amino acids (Romer and Scheibel, 2008; Vollrath, 2000) that may be targeted as substrate for microbes, therefore requiring antimicrobial properties as defense.

In the past decade, a range of studies have investigated antimicrobial properties of spider silk; however, results are conflicting with some reporting antimicrobial activity (AMA) (Al-Kalifawi and Kadem, 2017; Amaley et al., 2014; Keiser et al., 2015; Roozbahani et al., 2014; Tahir et al., 2017; Wright and Goodacre, 2012; Phartale et al., 2019; Deshmukh and Pansare, 2019), and others not (Zhang et al., 2019; Zortéa and Fischer, 2009; Babczynska et al., 2019; Alicea-Serrano et al., 2020; Szymkowiak et al., 2020). To assess the antimicrobial properties of spider silk in the light of contradictory evidence, we conducted a systematic test of AMA of different silk types from seven species covering the spider phylogeny. We tested different silk types for AMA against Gram-negative *Escherichia coli* and *Pseudomonas putida*, and Gram-positive *Bacillus subtilis*, using standardized direct contact or disc diffusion assays and fluorescence microscopy. Across all species and replicates, we found no evidence for AMA of silk of the studied spider species, i.e. no suppression of growth of any of the tested microbes.

A critical evaluation of studies that report AMA of spider silk revealed that the evidence in support of silk possessing intrinsic antimicrobial properties is compromised by methodological issues (Table 1). We detected two types of problems: (i) risk of bacterial contamination (Wright and Goodacre, 2012; Al-Kalifawi and Kadem, 2017; Amaley et al., 2014; Roozbahani et al., 2014; Tahir et al., 2017; Deshmukh and Pansare, 2019) and (ii) lack of control for solvent effects (Wright and Goodacre, 2012; Keiser et al., 2015; Tahir et al., 2017; Deshmukh and Pansare, 2019). Nevertheless, the notion of AMA of spider silk has been propagated by review papers (Romer and Scheibel, 2008; Saravanan, 2006) that have conveyed the message of AMA of spider silk based on flawed empirical studies, i.e. they have attributed AMA to silk citing empirical studies that do not contain evidence for AMA of spider silk (Table 1).

**Table 1:** Overview of published studies that assess antimicrobial activity (AMA) of spider silk and their findings. We include a brief note on the methodology used and evaluate whether there are appropriate controls of extraction method, and control for contamination, as decisive factors in drawing conclusion on AMA.

References	Bacteria (fungi)	Spider species	Findings	Method	Evaluation
Wright (2011)	*Bacillus subtilis* *Escherichia coli* (*Aspergillus niger*) (*Saccharomyces ceravisiae*)	*Araneus diadematus* *Lasiodora parahybana* *Pityohyphantes phrygianus* *Tegenaria domestica* *Zilla diodia*	Suggest that silk from *T. domestica* has AMA against *B. subtilis*	OD measurements of media with/without silk. Silk samples were either used natively, rinsed with water, subjected to UV light, or treated with Proteinase K. Disk diffusion assay (DDA) on agar plates of native silk	Large variation in reported OD for controls could suggest inconsistencies in the experimental setup. The Thesis (Wright, 2011) from which the work is published reports that samples were highly contaminated, and it was assumed by the author, that the contaminants did not influence the growth of the test organisms. Lack of control for contamination, AMA cannot unequivocally be attributed to silk
Wright and Goodacre (2012)	*B. subtilis* *E. coli*	*Tegenaria domestica*	Reports AMA against *B. subtilis*. Suggests weak AMA against *E. coli*	OD measurements of media with/without silk. Silk samples were either used natively, rinsed with water, subjected to UV light, or treated with Proteinase K	Results originate from Wright (2011) where it is reported that silk samples were highly contaminated. Unclear if replicates of OD measurements for each sample were performed. Lack of control for contamination. AMA cannot unequivocally be attributed to silk
Amaley et al. (2014)	*E. coli* *Klebsiella pneumoniae* *P. aeruginosa* *Staphylococcus aureus*	*Nephila pilipes*	Reports AMA against *E. coli*, *S. aureus*, *P. aeruginosa*	DDA of native silk samples	It is unclear how dragline silk was obtained and how it is identified to species. No report on measures to minimize risk of contamination. Four images of the DDA presented are of poor quality: silk is black but is expected to have a yellow taint and it is difficult to identify inhibition zones. Lack of control for contamination, AMA cannot unequivocally be attributed to silk
Roozbahani et al. (2014)	*E. coli* *Listeria monocytogenes*	*Pholcus phalangioides*	Reports AMA against *E. coli* and *L. monocytogenes* with DCT and WDA	500 mg of silk dissolved in 1% Tween 80 and 5% acetone for 60 days at room temperature then filtered. Extracts were examined with DCT and WDA	A silk sample is wrapped around a toothpick (not reported sterile), then placed on an agar plate with *L. monocytogenes* showing an uneven inhibition zone. For the WDA no results are shown. Lack of control for contamination. AMA cannot unequivocally be attributed to silk
Keiser et al. (2015)	*Bacillus thuringiensis*	*Stegodyphus dumicola*	AMA suggested with DDA	Filter discs wrapped in silk, dipped in 100% ethyl acetate and placed directly on agar plates show increased inhibition zones compared to controls Filter disks dipped in vortexed silk solution (100% ethyl acetate) do not increase inhibition zones	The discs may absrob different amounts of 100% ethyl acetate, discussed as a source of uncertainty. Control of the antimicrobial effect of 100% ethyl against *B. thuringiensis* is lacking (ethyl acetate is effective at killing *B. subtilis*, see Results). The reported differences in the size of inhibition zones could originate from the different uptake of 100% ethyl acetate on silk wrapped disc samples vs control. AMA cannot unequivocally be attributed to silk
Al-Kalifawi and Kadem (2017)	*B. subtilis* *Enterobacter cloacae* *E. coli* *K. pneumonia* *Pseudomonas aeruginosa* *Proteus mirabilis* *S. aureus* *Streptococcus spp*	*Tegenaria domestica*	Reports AMA against all test bacteria by acetone extracts of silk	Silk was washed in water, then incubated in water, ethanol, or acetone at 30°C for 7 days. Extracts were examined with WDA	Silk samples should originate from *T. domestica*, however the image of a web sample resembles an orb web where *T. domestica* produces funnel webs. The web samples and extracts are dark/grey, which suggests contamination. Some of the reported assay images reveal uneven bacterial growth. Lack of control for contamination. AMA cannot unequivocally be attributed to silk
Tahir et al. (2017)	*Acinetobactor sp.* *Streptococcus sp.*	*Cyclosa confraga*	AMA reported with DDA	50 mg orb web (unknown age) was collected on sterile glass rods and incubated in 100 mL 2.5% NaOH (incubation time not reported). Extracts were examined with DDA	Information on silk condition and sterility is lacking. Samples and controls were loaded on different agar plates with large variation in bacteria lawn density (control NaOH = high bacteria lawn density, samples = low density). Reported AMA could originate from the difference in bacteria lawn densities
Deshmukh and Pansare (2019)	*Escherichia coli Staphylococcus aureus*	*Stegodyphus sarasenorum*	AMA reported with DDA	Silk was washed in distilled water and oven dried, extracts prepared in ethanol, methanol or acetone	Silk collected in the wild from different loctions. AMA tests of solvents not provided. AMA cannot unequivocally be attributed to silk
Phartale et al., 2019	*Bacillus megaterium* *Klebsiella pneumonia* *P. aeruginosa* *Proteus vulgaris* *Staphylococcus aureus* *Salmonell typhi* (*Aspergillus niger*) (*A. flavus*) (*Candida albicans*) (*Ustilago maydis*) (*Alternaria solani*) (*Mucor hiemalis*)	*Pardosa brevivulva*	AMA reported by growth inhibition of *B. megaterium*, *S. typhi*, *and K. pneumoniae* by silk dissolved in formic acid and confirmed in DMSO fraction	Silk extracts were tested for AMA with DDA Several different extraction solvents were used, including chloroform, formic acid, ethanol, methanol, water, 1N HCl AMA reported for formic acid extracts/DMSO fraction	The species is a wandering spider with no capture web. Silk collected of plants in the field, spider species indentification not confirmed. No report of subsequent sterilization of silk. Formic acid extract of silk reported to show AMA but data not provided, and no control of formic acid solvent included. Subsequent DMSO fraction shows AMA with control for DMSO. Lack of control for contamination and formic acid solvent, AMA cannot unequivocally be attributed to silk
Zortéa and Fischer (2009)	*E. coli* *P. aeruginosa* *S. aureus* *Staphylococcus epidermidis* (*A. flavus*) (*Penicillum sp.*)	*Loxosceles intermedia* *Loxosceles laeta*	No AMA detected	Spiders were kept under sterile conditions and retreat silk collected AMA was investigated by WDA and DDA with native silk, water, and ethanol extracts	Appropriate controls
Zhang et al. (2019)	*Bacillus altitudinis* *B. subtilis* *Enterobacter bugandensis* *E. coli*	*Cyrtophora moluccensis* *Hippasa holmerae* *Nephila pilipes*	No AMA detected	AMA was investigated by growing bacteria in various media directly on orb webs and by cross-streaking (similar to disc diffusion assay)	Appropriate controls
Alicea-Serrano et al. (2020)	*E. coli*	*Latrodectus hesperus*	No AMA detected	AMA test in liquid *E. coli* culture	Risk that 100 silk treads of 5 mm length would not be sufficient to detect AMA
Szymkowiak et al. (2020)	*Escherichia coli*, *Pseudomonas aeruginosa*, *Staphylococcus aureus*, *Enterococcus faecalis*	*Linothele fallax L. mega-theloides*	No AMA detected	Silk sterilized with vaporized hydrogen peroxide, and added to Mueller-Hinton Broth. OD measurements used to test for AMA.	Attempts to avoid contamination. Positive controls includeded
Schulz and Toft (1993)	–	*Linyphia triangularis*	Identify ether lipids as a new class of natural products in spider silk	AMA of silk was not investigated	Speculative proposition that the lipid could play a role in protection against microbes
Saravanan (2006)	–	–	Review paper. Suggests AMA of silk based on Schulz and Toft (1993)	–	Conclusion not supported by direct evidence from Schulz and Toft (1993). See comment above
Hu et al. (2007)	–	*Latrodectus hesperus*	Identify metal chelators in silk (spider coating peptides)	AMA of silk was not investigated	It is speculated that the metal chelators function as a peptide chelator, which releases metal ions in response to pH changes to inhibit microbial growth
Romer and Scheibel (2008)	–	–	Review paper. Suggests AMA of silk based on Hu et al. (2007)	–	Conclusion not supported by direct evidence from Hu et al. (2007). See comment above

DCT, direct contact assay; DDA, disc diffusion assay; WDA, Well diffusion assay; OD, Optical density measurements.

## Results

### Tests of AMA

The prevailing method for testing AMA of spider silk has been to perform tests in the form of diffusion assays, where untreated silk (direct contact assay) or silk extracts (disc diffusion assay) are placed on agar plates inoculated with test bacteria. If a zone of inhibition appears on the test plate it is indicative of AMA (example in Figure 1). We assessed the putative antimicrobial effect of different silk types from seven spider species (Table 2) in direct contact (Table S2) or disc diffusion assays (Table S3) against three test bacteria; *Escherichia coli*, *Bacillus subtilis*, and *Pseudomonas putida*. AMA was never detected in any of the silk types or spider species tested (representative results are shown in Figure 1). These results were corroborated by epifluorescence microscopy of live/dead stained cells in contact with silk, showing that the majority of cells in direct contact with silk strands remained alive (Figure 1E). This was the case for all three test bacteria exposed to silk from *A. bruennichi* for 3 h, and exposed to silk from the other six spider species for 24 h.

**Table 2:** Summary of spider species and silk types used for testing of putative antimicrobial activity by direct contact assay, disc diffusion assays, or Live/dead stain of bacteria on silk strands. No antimicrobial activity was detected in any of the tests. Numbers are biological replicates.

Species	Silk type	Direct contact^a^	Disc diffusion	Live/dead stain^a^
***Argiope bruennichi*** (Araneidae) 4 individuals	Dragline	3		3
	Egg case silk	2		
***Araneus diadematus*** (Araneidae) 3 individuals	Dragline	3		1
	Orb web	3		
***Caribena versicolor*** (Theraphosidae) 2 individuals	Burrow web	2	2^b^	
***Latrodectus geometricus*** (Theridiidae) 6 individuals	Cob web	3		
	Egg case silk	5		
***Nephila edulis*** (Araneidae) 6 individuals	Dragline	4	Pool from 4 individuals^b^	2
	Orb web	5	Pool from 6 individuals^b^	
	Egg case silk	3		
***Stegodyphus dumicola*** (Eresidae) 3 colonies with >50 individuals in each	Dragline	4		2
	Nest web	3 colonies		3
	Capture web	3 colonies		
	Egg case silk	6		
	Nest + capture silk		2 x pool of 25 individuals^c^	
***Tegenaria domestica*** (Agelenidae) 4 individuals	Funnel web	3		2

a Numbers are number of biological replicates. Each replicate was tested against all three test strains.

b Extractions were made in both acetone and sodium hydroxide and incubated for 3 or 7 days.

c Extracted in ethyl acetate for 7 days.

Some of the most common solvents applied for AMA tests of silk extracts in the literature include acetone (Al-Kalifawi and Kadem, 2017; Roozbahani et al., 2014), sodium hydroxide (Tahir et al., 2017), and ethyl acetate (Keiser et al., 2015) (Table 1), and we assessed the bactericidal effect of these solvents. Silk from three spider species (*C. versicolor*, *N. edulis*, and *S. dumicola*) was extracted in these three solvents and tested for AMA in disc diffusion assays, including no-silk controls of solvent. We did not detect any differences in inhibition zones between silk extracts and no-silk controls (Figures 1B acetone, 1C sodium hydroxide, and 1D ethyl acetate), indicating that the inhibition zones detected result from bactericidal effects of solvents (Gómez-García et al., 2019) and not from the spider silk.

### Test of AMA of solvent

**Figure 2:**
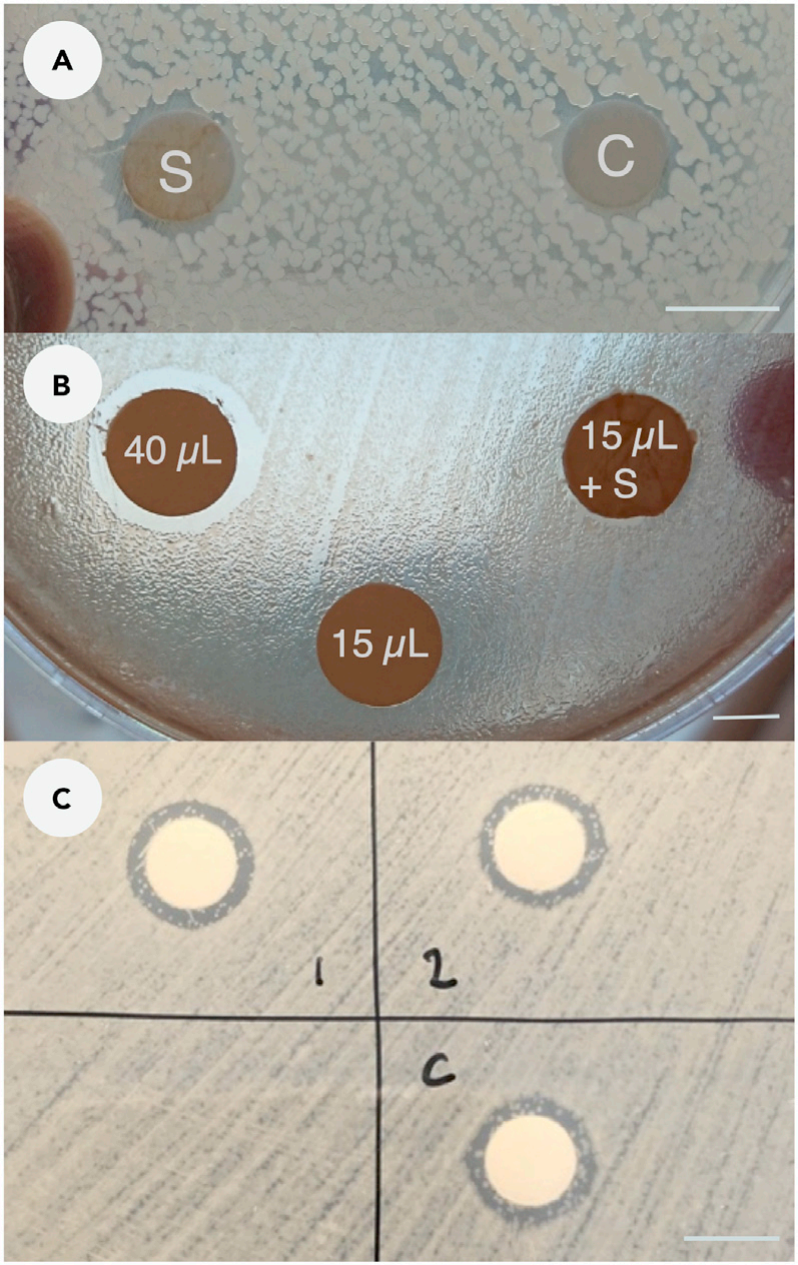
Test of antimicrobial activity of solvent: effect of 100% ethyl acetate (A) filter discs either wrapped in *S. dumicola* capture web silk (S) or control (C) were dipped in 100% ethyl acetate and placed on a test plate with *B. subtilis*. The filter wrapped in silk produced a slightly larger zone of inhibition, most likely due to a larger volume of absorbed solvent. (B) 100% ethyl acetate was pipetted directly onto filter discs; 40 μL, 15 μL ethyl acetate, and 15 μL ethyl acetate pipetted onto a filter disc wrapped in *S. dumicola* capture web silk (15 μL + S). (C) Silk extracted in 100% ethyl acetate for 7 days; 20 μL of two replicate extracts were pipetted onto filter discs (1, 2) and 20 μL pure 100% ethyl acetate was used as a control.

Keiser et al. (2015) reported AMA from *S. dumicola* capture web dipped in 100% ethyl acetate, and we replicated this procedure to determine the amount of solvent adhering to the filter discs after dipping. We found that control filter discs absorbed 43 ± 4 mg ethyl acetate when dipped in the solvent, whereas filters with silk wrapped around absorbed more than three times that amount of solvent (142 ± 25 mg, Table S1), and consequently produced a larger inhibition zone (Figure 2A). Inhibition zones from 40 μL ethyl acetate pipetted directly onto a filter disc were much larger than inhibition zones produced from 15 μL ethyl acetate (Figure 2B). When 15 μL ethyl acetate was pipetted onto filter discs wrapped in silk, the silk strands drew the liquid closer to the edge of the filter disc, resulting in a slightly larger zone of inhibition compared with the control (Figure 2B). To further investigate if putative antimicrobial compounds could indeed be extracted from the silk, we submerged clean silk in 100% ethyl acetate for 7 days. These extracts were subsequently used in a disc diffusion assay (Figure 2C), and we found no difference in the zone of inhibition between the solvent control and the two replicate silk extracts.

## Discussion

We tested the AMA of different silk types originating from seven spider species and found no evidence for growth inhibition of three test bacteria *E. coli*, *B. subtilis* and *P. putida*. These bacteria were selected as they are frequently used in assays of AMA, and contrasting results were previously reported for some combinations of spider species and bacteria (Table 1). Furthermore, images from fluorescence microscopy (live/dead staining) showed live bacteria present on dragline silk surfaces, corroborating the data from direct contact assays showing that silk does not possess AMA.

Several studies reported that spider silk from specific species did not show antimicrobial properties: Zortéa and Fischer (2009) investigated the AMA of retreat silk from *Loxosceles laeta* and *L. intermedia* and found no bacterial or fungal inhibition when testing sterile silk directly or in water or ethanol extracts. Zhang et al. (2019) inoculated several types of bacteria directly on silk from the spiders *Nephila pilipes*, *Hippasa holmerae,* and *Cyrtophora moluccensis* but found no evidence for inhibition. Alicea-Serrano et al. (2020) assessed AMA of dragline silk of the spider *Latrodectus hesperus* in an aqueous *Escherichia coli* cell culture and found no evidence for antimicrobial properties, and Szymkowiak et al. (2020) found no evidence for AMA in two *Linothele* species in OD measurements of silk extracts inoculated with four different bacteria. The methodologies applied in these studies include different test species, and a variety of types of assays and solvents, it is therefore unlikely that systematic methodological effects compromises the results.

Because silk mainly consists of proteins and small quantities of carbohydrates in the form of glycosylated proteins in the thin outer shells, it has been hypothesized that bacteria and fungi could use silk as a food source (Zhang et al., 2019). However, as the core silk spidroin structure is water-insoluble and partially packed as crystalline β-sheets, it is not expected to be an easily degradable food source and could require specialized digestion enzymes for degradation. These properties are consistent with the hypothesis that β-silk sheets contributes to providing a physical barrier to microbes. In support of this idea, Babczynska et al. (2019) found that silk cases of the spider *Parasteatoda tepidariorum* placed on broth agar did not inhibit bacterial growth, i.e. showing no antimicrobial effect, whereas in contrast, no bacterial growth was visible when eggs were taken out of the egg case and directly incubated on broth agar. This indicates that the eggs inside the egg case are sterile, implying that the egg case provides a physical barrier to protect the eggs (Babczynska et al., 2019). Interestingly, widow spider (*Latrodectus geometricus*) eggshells were found to possess properties that protect the eggs from bacterial infection (Makover et al., 2019). Spider egg cases are made of silk that is tightly woven into sheets of fabric several layers thick, as illustrated with SEM images of *S. dumicola* egg cases (Figures S2 and S3). It is therefore possible that the main protection offered by egg case silk is in the form of a physical barrier (Babczynska et al., 2019).

Spider silk is recognized as a biomaterial with unusual and potentially highly useful properties with relevance for different applications (Vepari and Kaplan, 2007). In relation to medical applications to combat infections, there is currently focus on taking advantage of the extraordinary properties of spider silk, which makes it suitable for bioengineering of antimicrobial silk by fusing spider silk with synthesized antimicrobial peptides (Franco et al., 2018; Kumari et al., 2020; Chouhan and Mandal, 2020).

Antimicrobial peptides have been reported in glands from insects, most prominently in silkworms (Yi et al., 2014), and it was therefore suggested that these antimicrobial proteins provide antibacterial properties to silk cocoons. However, a study suggests that reports of antimicrobial properties of cocoon silk in fact results from residues of chemicals that were used to isolate or purify cocoon elements (Kaur et al., 2014). Indeed, they show that “properly isolated” silk fiber, gum, and embedded crystals free from chemical residues do not have inherent resistance to the test bacterium *E. coli* (Kaur et al., 2014). While this does not necessarily preclude the existence of AMA of silkworm silk, it emphasizes the need to apply appropriate methodologies and controls in the study of AMA of natural materials such as silk. Application of silkworm silk for medical purposes, similar to spider silk, therefore appears to rely on bioengineering or genetic engineering of modified silkworm strains with antimicrobial properties of silk (e.g. (Saviane et al., 2018)).

Next, we discuss reports of AMA of spider silk in light of methodological shortcomings identified, including: (i) risk of bacterial contamination (Wright and Goodacre, 2012; Al-Kalifawi and Kadem, 2017; Amaley et al., 2014; Roozbahani et al., 2014; Tahir et al., 2017; Phartale et al., 2019), (ii) lack of control for solvent effects (Wright and Goodacre, 2012; Keiser et al., 2015; Tahir et al., 2017; Deshmukh and Pansare, 2019), and (iii) reviews (Romer and Scheibel, 2008; Saravanan, 2006) conveying statements of AMA of silk based on inadequate evidence (Table 1).

Wright and Goodacre (2012) investigated AMA in webs from *Tegenaria domestica* and reported inhibition of growth of *B. subtilis*. However, false positives could be due to contamination. Their data originate from an MSc thesis (Wright, 2011) in which silk samples were reported to be contaminated with unknown bacteria and fungi. It was not investigated whether these contaminants had AMA. This precludes the separation of intrinsic antimicrobial properties of the spider silk, from that of contamination of bacteria with possible AMA against *B. subtilis*.

Other positive reports of AMA on spider silk (Al-Kalifawi and Kadem, 2017; Amaley et al., 2014; Roozbahani et al., 2014; Deshmukh and Pansare, 2019) may also result from web contamination, as webs of unknown age (and sometimes from unknown species) were investigated, and images of silk samples in some cases indicate severe contamination. Acetone extraction provided the best inhibition according to these articles, however, whether inhibition originated from contamination rather than from the silk was not examined. To investigate this problem, we produced acetone extracts of sterile fresh web and dragline silk but found no increased inhibition zone compared to the negative controls (Table S3).

Keiser et al. (2015) concluded that clean *S. dumicola* nest and capture web silk weakly inhibits the growth of *Bacillus thuringiensis* (Table 1). However, their method of dipping silk-wrapped filter paper discs and controls (plain filter discs) into 100% ethyl acetate (CH_3_COOCH_2_CH_3_) is liable to create large variation in solvent volume uptake (Table S1). This is problematic as ethyl acetate effectively inhibits bacterial growth (tested against *Pseudomonas aeruginosa*, *Staphylococcus aureus*, *E. coli*, *Candida albicans,* and *Trichophyton rubrum*) with a minimum inhibitory concentration of <5% (Lens et al., 2016). Although the paper reports that “preliminary experiments suggest that ethyl acetate itself does not have antibacterial activity against *B. thuringiensis* (unpubl. data)”, they did not specify the procedure and did not include the data to support this observation. We tested the effect of ethyl acetate on bacterial growth and show that 100% ethyl acetate is lethal for *B. subtilis* (Figure S1). In our setup, silk samples dipped in 100% ethyl acetate displayed larger inhibition zones than controls for the three bacteria *E. coli*, *B. subtilis* and *P. putida*. However, when equal volumes of 100% ethyl acetate were pipetted onto silk samples and controls, inhibition zones were similar in size between controls and silk samples (Figures 2B and 2C). Therefore, the difference in inhibition zones between silk samples and controls reported in Keiser et al. (2015) most likely result from larger volume uptake of 100% ethyl acetate by silk samples (Table S1) than from AMA of silk.

Sodium hydroxide extraction of *Cyclosa confraga* silk was proposed to inhibit *Streptococcus* sp. and *Acinetobacter* sp (Tahir et al., 2017). However, sodium hydroxide control plates had high bacteria lawn density and a small inhibition zone, whereas silk sample plates had low bacteria lawn density and a large inhibition zone. To eliminate this problem, controls and samples should be placed on the same agar plate to provide identical conditions allowing for precise comparison. Our tests of sodium hydroxide extract showed same or smaller size inhibition zones between silk samples and control (Figure 1C), suggesting no inhibition caused by spider silk.

Phartale et al., 2019 tested AMA of *Pardosa brevivulva* silk (although origin of silk not verified) against several gram-positive and gram-negative bacterial strains, as well as against test fungi. Using formic acid extracts of silk, they claim growth inhibition of the bacteria *Bacillus megaterium*, *Salmonella typhi*, and *Klebsiella pneumonia*, and antifungal potential by the inhibition of the fungi *Aspergillus flavus*, *Candida albicans*, *Ustilago maydis*, and *Alternaria solani*, however without providing any supporting evidence. Instead the paper shows results of AMA of DMSO fractions against *B. megaterium*, *S. typhi*, and *K. pneumonia*. There are two main concerns: risk of contamination as spider silk was collected from plants in the field with no report of subsequent sterilization, and the lack of control for AMA of the formic acid solvent, as formic acid shows effective antibacterial activity (Gómez-García et al., 2019). The DMSO fraction of formic acid silk extract would retain diluted solvent. Phartale et al., 2019 performed subsequent chemical analyses of “bioactive” silk and did not identify antimicrobial molecules.

A review by Saravanan (2006) states that: “Presence of 12-methyltetradecanoic acid and 14-methylhexadecanoic acid to the minor amounts impart antimicrobial properties to the spider silk”. This statement is based on the presence of these chemicals in silk from *Linyphia triangularis* (Schulz and Toft, 1993), and not on specific tests of AMA of spider silk. Hattori et al. (1987) investigated growth inhibition of various fatty acids against *Streptococcus mutans*, and 12-methyltetradecanoic acid and 14-methylhexadecanoic acid were found to inhibit growth at a Minimum Inhibitory Concentration of 3.13 μg/mL. There is no test showing whether silk of *L. triangularis* is antimicrobial or inhibits bacteria growth, and if so, whether these fatty acids are responsible.

The review of Romer and Scheibel (2008) conveys the general notion of AMA of spider silk. However, the only reference to AMA is a study (Hu et al., 2007) reporting small peptides SCP-1 and SCP-2 in aggregate glue on silk of *Latrodectus hesperus*. These peptides were hypothesized to function as metal chelators, and it was further speculated that release of metal ions from these peptides could have antimicrobial function. However, this has not been verified. Our test of AMA of silk from the congener *Latrodectus geometricus* shows no AMA and therefore does not corroborate their hypothesis.

In summary, the evaluation of empirical data suggests that reported results of AMA of spider silk are compromised by inadequate controls for contamination or for bactericidal effects of solvents, and consequently, the observed antimicrobial inhibition cannot be unequivocally attributed to spider silk. Overall, therefore, the results presented here combined with a critical assessment of existing data strongly refutes the existence of intrinsic antimicrobial properties of spider silk. Naturally, we cannot exclude the possibility that silk of other and so far untested spider species might show antimicrobial properties, or that spider silk may show AMA against microbes that were not tested in the studies reported here. Nevertheless, the current evidence shows that the widely held expectation of inherent antimicrobial properties of spider silk is not justified.

### Conclusions

Assays of AMA of different types of spider silk from seven species provided no evidence that silk possesses AMA. A critical evaluation of studies that assesses AMA of spider silk revealed methodological shortcomings that compromise previous reports of AMA of silk. Collectively, our data and the identified shortcomings of empirical studies of spider species studied thus far strongly refute the notion of intrinsic antimicrobial properties of spider silk. Some evidence exists that egg case silk may provide structural protection by forming a barrier against pathogens.

### Limitations of the study

The conclusions of the study are based on tests of seven different spider species, in addition to a critical evaluation of existing reports of antimicrobial properties of spider silk. We cannot exclude the possibility that silk of other and so far untested spider species might show antimicrobial properties, or that spider silk may show AMA against other microbes that were not tested in the studies reported here.

## STAR★Methods

### Key resources table

**Table:** 

REAGENT or RESOURCE	SOURCE	IDENTIFIER
**Bacterial and virus strains**		

*Escherichia coli*	DSMZ	DSM498
*Bacillus subtilis*	DSMZ	NCIB 3610
*Pseudomonas putida*	DSMZ	DSM6125

**Biological samples**		

Spider silk	*Argiope bruennichi*	
	*Araneus diadematus*	
	*Caribena versicolor*	
	*Latrodectus geometricus*	
	*Nephila edulis*	
	*Stegodyphus dumicola*	
	*Tegenaria domestica*	

**Chemicals, peptides, and recombinant proteins**		

ethyl acetate		
sodium hydroxide		
acetone		

### Resource availability

#### Lead contact

Requests for further information should be directed to Trine Bilde (trine.bilde@bio.au.dk).

#### Materials availability

This study did not generate new unique material or reagents.

### Experimental model and subject details

#### Spider collection and rearing

Adult females of *Araneus diadematus* (3 individuals), *Argiope bruennichi* (4 individuals), and *Tegenaria domestica* (4 individuals) were collected from the wild in the vicinity of Aarhus, Denmark in spring 2018 and kept in clean plastic containers of 15 × 15 × 7 cm. Nests of the social spider *Stegodyphus dumicola* were collected in Botswana and transported to Denmark. Colonies of about 30 females (a mixture of sub adults and adults) were kept in clean plastic containers of 10 × 10 × 18 cm.

Six adult female *Latrodectus geometricus* were collected in the Negev Desert, Israel in spring 2018. Upon arrival to Aarhus they were placed in clean plastic containers of 30 × 30 × 43 cm with thin wooden sticks glued to the sides to aid web attachment. Six adult female *Nephila edulis*, originating from a lab colony at Oxford University, were transferred to Aarhus University in 2018 and kept in wooden boxes of 50 × 50 × 15 cm with acrylic fronts and backs. Two female *Caribena versicolor* were obtained from a private supplier in 2015. They were moved to individual clean glass containers for production of new clean burrows, which took 1 month.

All spiders, except *S. dumicola*, were kept at room temperature (about 21°C) with a relative humidity (RH) of about 70%. *Stegodyphus dumicola* were kept in a temperature-controlled room at 29°C with varying RH between 60-90%. All spiders were watered every second day with a spray bottle and they were fed twice a week with small flies (*Lucilia* obtained from Peter Andersen Aps). *Latrodectus geometricus* and *S. dumicola* were additionally fed with small crickets (*Gryllus bimaculatus*). *Caribena versicolor* were not fed flies, but were given a diet of adult crickets, grasshoppers (*Locusta migratoria* obtained) and cockroaches (*Blaptica dubia* from a lab culture).

#### Cultivation of test organisms

*Escherichia coli* (DSM 498), *Bacillus subtilis* (NCIB 3610) and *Pseudomonas putida* (DSM6125) were cultivated from -80°C glycerol stock on NB agar plates (8 g/L Nutrient Broth (APHA, Scharlau Microbiology), 12 g/L agar, Scharlau Microbiology). *E. coli* and *B. subtilis* were incubated at 35°C and *P. putida* at 30°C. All experiments were inoculated from 6 mL overnight starter cultures grown in 12 g/L NB medium in Falcon tubes and diluted with NB medium to the desired optical density (OD) at 600 nm.

### Method details

#### Collection of silk

Dragline silk from *A. bruennichi*, *A. diadematus*, *N. edulis*, and *S. dumicola* was reeled out of immobilized spiders at a constant rate of 3 cm/s, by a mechanical LEGO setup (see Figure S4). 1-2 mg of dragline was formed into small discs and placed upon test plates by sterile forceps. Orb webs from *A. diadematus* and *N. edulis*, and upper parts of cob web from *L. geometricus* were collected using sterile scissors and forceps. Webs were collected shortly after production to minimize contamination and placed directly onto test plates. Funnel web (*T. domestica*), burrow web (*C. versicolor*) and nest web (*S. dumicola*) were collected to form circular discs (Ø 5 mm). The three species were transferred to clean enclosures and not fed or watered until after collection of silk (2-7 days for *T. domestica and S. dumicola*, ∼30 days for *C. versicolor*) to minimize contamination. Visible contamination, such as exoskeletons from moulting, was removed from the silk when present.

Spider enclosures were checked for egg sacs every third day and investigated on the day of collection, except for *N. edulis,* eggs sacs that were stored for 6 months at 4°C. Eggs were removed from the egg sacs and the egg case silk was placed on test plates. For *A. bruennichi* the inner and outer silk could be separated and was investigated individually. *Stegodyphus dumicola* and *L. geometricus* egg sacs were cut open and placed with either outer side (outer silk) or inner side (inner silk/ovulation fluid) facing the agar.

#### Preparation of silk extracts

Fresh dragline silk (approximately 10 mg), newly produced orb web silk (approximately 100 mg) from four *N. edulis*, and burrow web (approximately 50 mg) from two *C. versicolor* was collected as described above. Each sample was extracted in either 5 mL of 2.5% w/v NaOH or 1 mL 99.9% acetone for 3 or 7 days at 28°C rotating at 30 RPM. The remaining silk was removed, and solutions filtered through 0.22 μm filters (Sartorius). 35 μL of extract or control (2.5% w/v NaOH or 99.9% acetone to test for a potential antimicrobial effect of the solvent alone) was pipetted onto 6 mm cotton discs (Whatman Grade AA discs 6 mm) and placed on test plates and incubated overnight.

To obtain a sufficient amount of clean capture web and nest silk from *S. dumicola*, 25 individuals were placed in each of two clean plastic boxes allowing web production to take place for 10 consecutive days. The spiders were watered twice, but no food was given to limit contamination of the silk. The silk was harvested from the two replicate boxes (8.1 mg and 7.8 mg) and submerged in 100 μL pure ethyl acetate and incubated for 7 days at room temperature. For the disc diffusion assay, 20 μL extract or 20 μL solvent control (ethyl acetate) were pipetted directly onto filter discs (Whatman Grade AA discs 6 mm) on the test plates and incubated overnight.

If ethyl acetate has a toxic or antibiotic effect, the amount of solvent applied may influence the results by inhibiting bacterial growth. Furthermore, the amount of solvent absorbed could differ between silk-wrapped filter discs and control discs (Keiser et al., 2015). To assess whether this is the case, filter discs wrapped in *S. dumicola* capture web silk and control filter discs (no silk) were dipped in 100 % ethyl acetate. The weights of the filter discs were recorded before and after dipping, and the discs were incubated on test plates of all three test organisms overnight. To test if a larger volume of ethyl acetate would produce a larger zone of inhibition, a known volume of ethyl acetate was pipetted directly on to a filter disc, which was placed on a test plate; 15 and 40 μL were added to control filter discs, and 15 μL was added to a filter disc wrapped in *S. dumicola* capture web silk.

#### Direct contact and disc diffusion assays

NB agar plates used for silk diffusion tests were prepared one day prior to use to ensure similar water content in the plates. All three test strains were diluted to OD 0.10 and spread across the surface of NB agar plates using sterile cotton swaps. Samples were immediately placed on the test plates and incubated overnight. All tests were carried out against three test organisms (*E. coli*, *B. subtilis* and *P. putida*). Silk was collected from individual spiders, or from the silk nest of the social spider *S. dumicola*, and each silk sample was further divided in three subsamples to be tested against *E. coli*, *B. subtilis* and *P. putida* respectively. The numbers of individuals for each spider species and the silk type used in tests are provided in Table 2.

#### Live/dead stain of bacteria in contact with silk

*Argiope bruennichi* dragline silk was reeled manually at slow speed (3-6 cm/s) around cover slips (Menzel-Gläser 25 × 50 mm). The silk was held in place with sticky tape added to the edges of the cover slip and silk was removed from the opposite side to have a clear view for downstream microscopy. 250 μL of each test bacterium (OD 0.10) was pipetted on top of the silk and incubated in sterile Petri dishes at 35°C (*E. coli* and *B. subtilis*) or 30°C (*P. putida*) for 3h. Equal volumes of live/dead dyes (Invitrogen LIVE/DEAD Baclight Bacterial Viability kit, L7012) were mixed and 1 μL was slowly added to the bacteria overlaying the silk. The samples were incubated in the dark for 10 minutes at 21°C before imaging on an inverted epifluorescence microscope (Zeiss Axiovert 200M). This procedure was repeated with dragline silk from *N. edulis*, funnel web from *T. domestica*, and dragline and nest silk from *S. dumicola*, however, the incubation time with the three test organisms was increased to 24 hours. Droplets of sterile water was added along the edge of the Petri dish to increase the relative humidity and avoid evaporation from the test culture.

### Any additional information

No additional information is available.

### Quantification and statistical analysis

Information on detection of antimicrobial activity is provided in STAR Methods. No statistical analyses are applied.

## Data Availability

All data has been presented throughout the paper. This paper does not report original code.
