# Supplementary material for: The myth of antibiotic spider silk
Source: iScience. 2021 Oct 5;24(10):103125. doi: 10.1016/j.isci.2021.103125 (PMC8560547; doi:10.1016/j.isci.2021.103125)
Supplement: Document S1. Figures S1–S4 and Tables S1–S3 [file mmc1.pdf]

iScience, Volume *24*

Supplemental information

The myth of antibiotic spider silk

Simon Fruergaard, Marie Braad Lund, Andreas Schramm, Thomas Vosegaard, and Trine Bilde

Supplementary material

Figure S1. The antimicrobial effect of the solvent ethyl acetate against *B. subtilis,* incubated for 20h at 35 °C, see Figure 2 and STAR methods.
The antimicrobial effect of ethyl acetate was tested by dipping a sterile cotton swab in ethyl acetate and gently dragging it around on a *B. subtilis* lawn on an agar-media plate. A Control applying the same setup with LB media instead of ethyl acetate showed no inhibition.

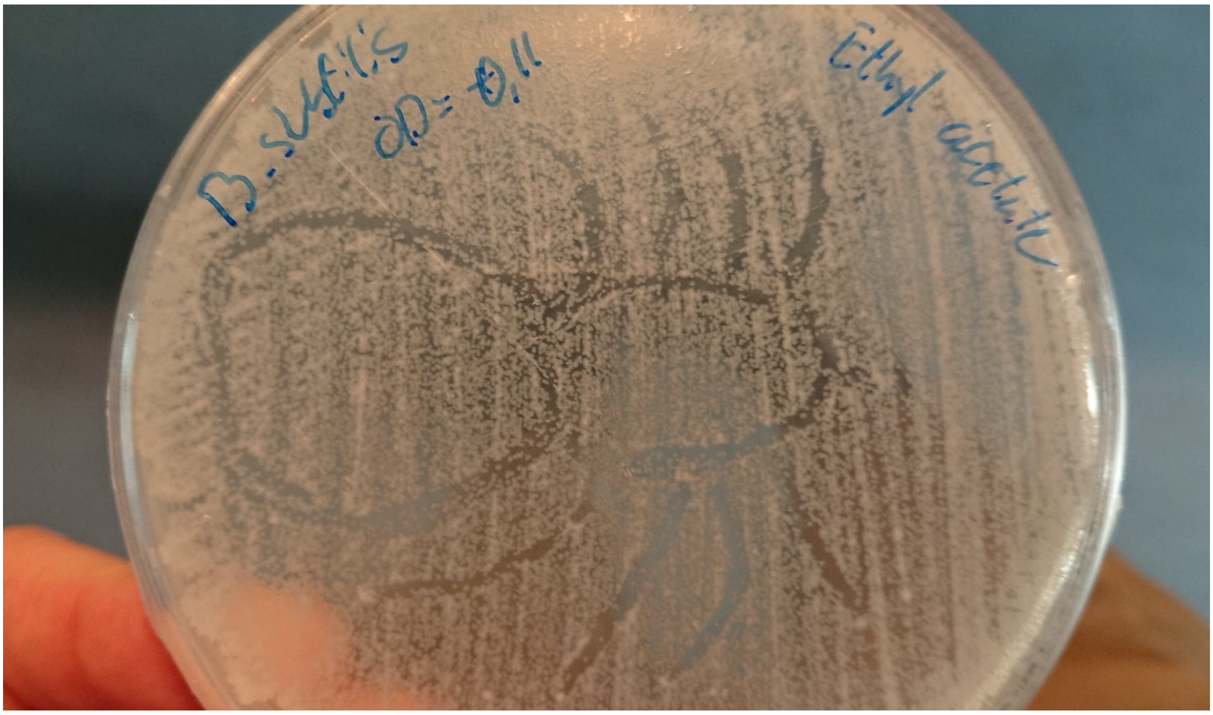

Figure S2. SEM images of *S. dumicola* outer surface of an egg case at two magnifications. The blue square in image A represent the area of image B. Scalebars are 200 µm (A) and 50 µm (B). See Figure 1 and see STAR methods for procedure.

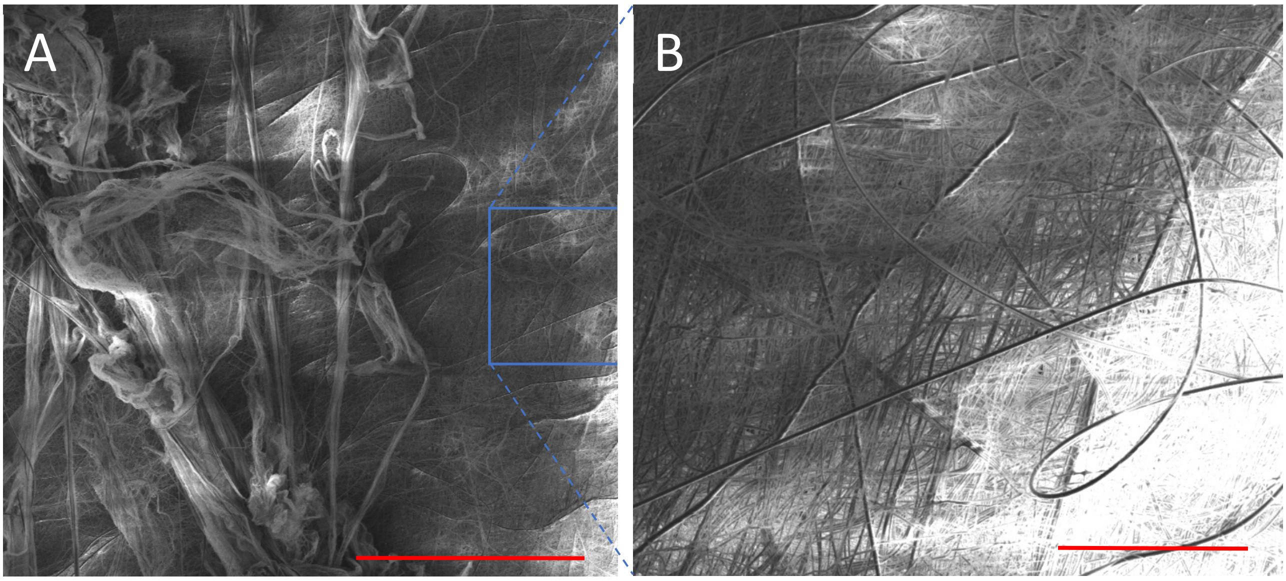

Figure S3. SEM image of *S. dumicola* egg case cross-section with an egg and an eggshell above the blue arrow. The blue arrow indicates the inner silk, aciniform, and the green arrow indicates the outer silk, cylindrical. Scalebar = 500 µm. See Figure 1 and see STAR methods for procedure.

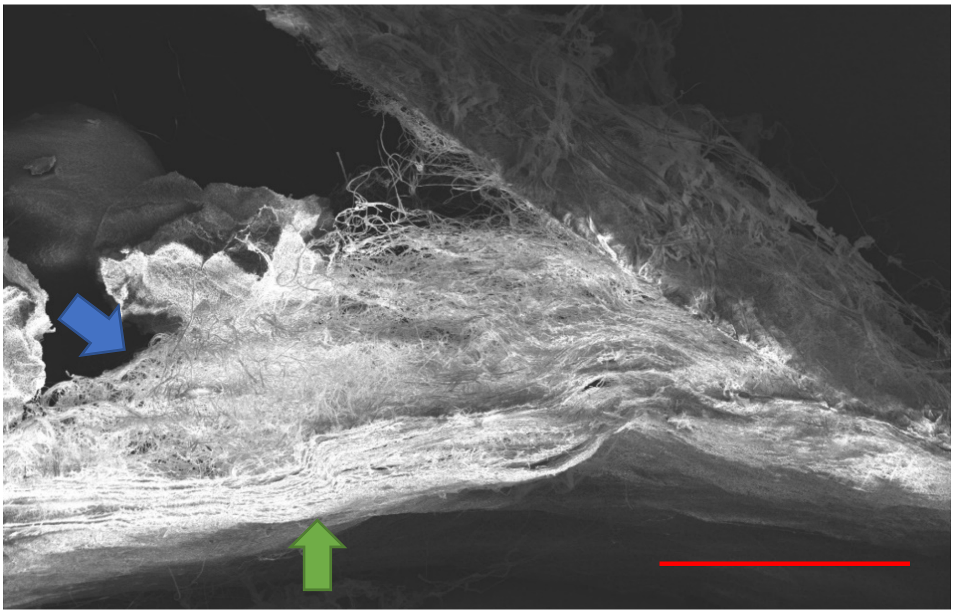

Figure S4. The mechanical LEGO setup utilized to reel out dragline silk from immobilized spiders (the spider *Nephila edulis* shown here). Relates to STAR methods.

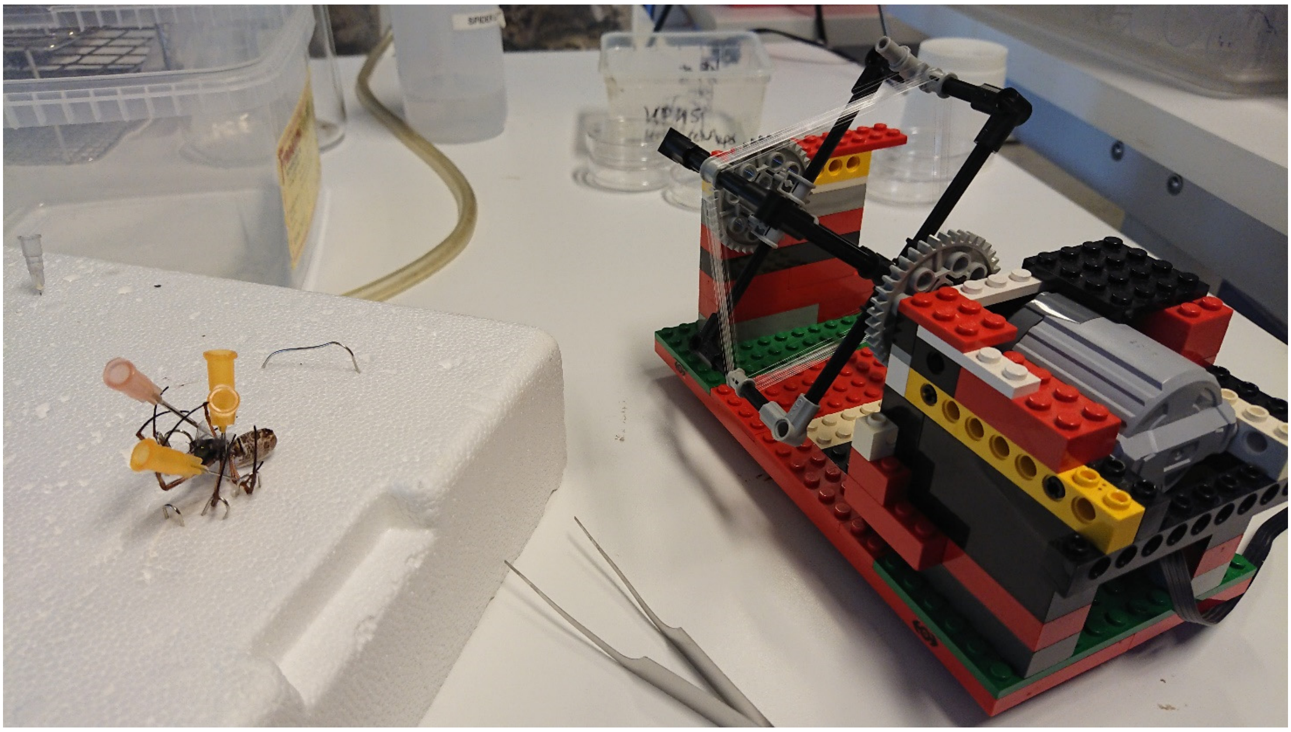

Table S1. Weight of filter discs before and after dipping in 100 % ethyl acetate, see Figure 2.

	Control filter disc (no silk)			Filter disc wrapped in *S. dumicola* capture web		
	Weight, mg			Weight, mg		
Sample	Dry	Wet	Increase	Dry	Wet	Increase
1	6.9	55	**48.1**	7.7	142	**134.3**
2	6.9	47	**40.1**	8.1	190	**181.9**
3	6.9	53	**46.1**	7.8	139	**131.2**
4	6.8	46	**39.2**	8.0	122	**114.0**
5	6.9	48	**41.1**	8.5	156	**147.5**

Table S2. Direct contact assays with untreated silk, relates to Figure 1 and STAR methods.

**Untreated silk**	***B. subtilis***	***E. coli***	***P. putida***
S. dumicola dragline	-	-	-
S. dumicola nest web	-	-	-
S. dumicola capture web	-	-	-
N. edulis dragline	-	-	-
N. edulis orb web	-	-	-
C. versicolor burrow web	-	-	-
A. diadematus dragline	-	-	-
A. diadematus orb web	-	-	-
A. bruennichi dragline	-	-	-
T. domestica funnel web	-	-	-
L. geometricus cob web	-	-	-
**Untreated egg sac silk**			
S. dumicola outer silk	-	-	-
S. dumicola inner silk	-	-	-
*N. edulis**	-	-	-
A. bruennichi outer silk	-	-	-
A. bruennichi inner silk	-	-	-
L. geometricus outer silk	-	-	-
L. geometricus inner silk	-	-	-

*Inner and outer silk types could not be distinguished

Table S3. Disc diffusion assays of silk extracted in sodium hydroxide or acetone. Relates to Figure 1.

	**Sodium hydroxide extract**			**Acetone extract**	
	B. subtilis	***E. coli***	***P. putida***	***B. subtilis***	***E. coli***
N. edulis dragline	+	+	+	+	-
N. edulis orb web	+	+	+	+	-
C. versicolor burrow web	+	+	+	+	-
Control	+	+	+	+	-
